# A Novel Efficient Graph Model for the Multiple Longest Common Subsequences (MLCS) Problem

**DOI:** 10.3389/fgene.2017.00104

**Published:** 2017-08-09

**Authors:** Zhan Peng, Yuping Wang

**Affiliations:** School of Computer Science and Technology, Xidian University Xi'an, China

**Keywords:** multiple longest common subsequences, longest common subsequence, dominant point method, directed acyclic graph, biological sequence alignment

## Abstract

Searching for the Multiple Longest Common Subsequences (MLCS) of multiple sequences is a classical NP-hard problem, which has been used in many applications. One of the most effective exact approaches for the MLCS problem is based on dominant point graph, which is a kind of directed acyclic graph (DAG). However, the time and space efficiency of the leading dominant point graph based approaches is still unsatisfactory: constructing the dominated point graph used by these approaches requires a huge amount of time and space, which hinders the applications of these approaches to large-scale and long sequences. To address this issue, in this paper, we propose a new time and space efficient graph model called the Leveled-DAG for the MLCS problem. The Leveled-DAG can timely eliminate all the nodes in the graph that cannot contribute to the construction of MLCS during constructing. At any moment, only the current level and some previously generated nodes in the graph need to be kept in memory, which can greatly reduce the memory consumption. Also, the final graph contains only one node in which all of the wanted MLCS are saved, thus, no additional operations for searching the MLCS are needed. The experiments are conducted on real biological sequences with different numbers and lengths respectively, and the proposed algorithm is compared with three state-of-the-art algorithms. The experimental results show that the time and space needed for the Leveled-DAG approach are smaller than those for the compared algorithms especially on large-scale and long sequences.

## 1. Introduction

Measuring the similarity of biological sequences is a fundamental problem in bioinformatics, which has many applications such as in cancer diagnosis (Aravanis et al., 2017) and detection of the species common origin (Zvelebil and Baum, 2007), etc. One of the most important ways to measure the similarity of sequences is to find their Longest Common Subsequences (LCS), which has been proved to be a NP-hard problem (Maier, 1978). According to the number of sequences, the problems are classified into two cases: (1) Looking for the longest common subsequence of *two* sequences is called the Longest Common Subsequence (LCS) problem. (2) Looking for the longest common subsequence of *more than two* sequences is called the Multiple Longest Common Subsequences (MLCS) problem.

Traditionally, the works are mainly focused on the first kind of problem. However, in recent years, more and more applications in bioinformatics (and many other fields) require to look for the longest common subsequence of many sequences. For example, one of the most important applications of the MLCS algorithms in bioinformatics is multiple sequence alignment (MSA), which is an essential technique of arranging the sequences of DNA, RNA, or protein to identify regions of similarity that may be a consequence of functional, structural, or evolutionary relationships between the sequences. The MLCS algorithms can also be used for other types of sequences, such as calculating the edit distance cost between strings in a natural language or in financial data. Although there are algorithms proposed for these applications, they are not efficient enough for many and long sequences due to their high time and space overhead.

In this paper, we propose a new time and space efficient graph model called the leveled directed acyclic graph (*Leveled-DAG* for short) and design the corresponding algorithm to construct it. The Leveled-DAG is constructed level by level, which is similar to the construction of DAG in the existing dominant point based algorithms, however, the existing dominant point based algorithms have to generate a huge number of the nodes and save them all in memory, while the Leveled-DAG approach can timely eliminate all the nodes in the graph that cannot contribute to the construction of MLCS any more. At any moment, only the nodes in the current level as well as some nodes in the previous levels are saved, therefore, the Leveled-DAG is much smaller than the DAG constructed by the existing dominant point based algorithms, which can save a lot memory space. Moreover, with the progress of the construction procedure, the scale of the Leveled-DAG becomes smaller and smaller, and the final graph contains only one node (the end node) with the wanted MLCS saved in it, thus, no operations to search for them are needed, which can save a lot of running time. The experimental results show that our approach is both time and space efficient for large-scale MLCS problems with real biological sequences and performs better than the compared leading dominant point based algorithms.

This paper is organized as follows: Section 2.1 introduces some preliminaries and reviews the related work. In Section 2.2, the Leveled-DAG model and its construction algorithm is presented in detail. In Section 3, we compare the performance of the proposed algorithm with that of the existing stat-of-the-art ones via experiments. At last, we summarize the paper.

## 2. Materials and methods

In Section 2.1, we will first introduce some preliminaries and related work about MLCS problem, and then in Section 2.2, the new Leveled-DAG model and the algorithm to construct it will be illustrated in detail.

### 2.1. Preliminaries and related work

First of all, let Σ denote the alphabet of the sequences, i.e., a finite set of symbols. For example, the alphabet of the DNA sequences is Σ = {*A, C, G, T*}.

**Definition 1**. Let Σ denote the alphabet and *s* = *c*_1_*c*_2_…*c*_*n*_ be a sequence of length *n* with each symbol *c*_*i*_ ∈ Σ, for *i* = 1, 2, ⋯ , *n*. The *i*-th symbol of *s* is denoted by *s*[*i*] = *c*_*i*_. If a sequence *s*′ is obtained by deleting zero or more symbols (not necessarily consecutive) from *s*, i.e., $s^{\prime }=c_{i_{1}}c_{i_{2}}\ldots c_{i_{k}}$ satisfying 1 ≤ *i*_1_ < *i*_2_ < ⋯ < *i*_*k*_ ≤ *n*, then *s*′ is called a length *k subsequence* of *s*.

**Definition 2**. Given *d* sequences *s*_1_, *s*_2_, …, *s*_*d*_ on Σ, if a sequence $s^{\prime }=c_{i_{1}}c_{i_{2}}\ldots c_{i_{k}}$ satisfies: (1) It is a subsequence of each of these *d* sequences. (2) It is the longest subsequence of these *d* sequences. Then *s*′ is called a Longest Common Subsequence (*LCS*) of these *d* sequences.

Generally, LCS of multiple sequences is not unique. For example, given three DNA sequences *ACTAGTGC*, *TGCTAGCA* and *CATGCGAT*, there exists two LCSs of length 4, which are *CAGC* and *CTGC*, respectively. The multiple longest common subsequences (MLCS) problem is to find all the longest common subsequences of three or more sequences.

Many algorithms have been proposed for the MLCS problem in the past decades. According to the models on which the algorithms are based, the existing MLCS algorithms can be classified into two categories: the dynamic programming based approaches and the dominant point based approaches. Next, we will give an brief introduction to each of the two approaches.

#### 2.1.1. Dynamic programming based approaches

**Figure 1:**
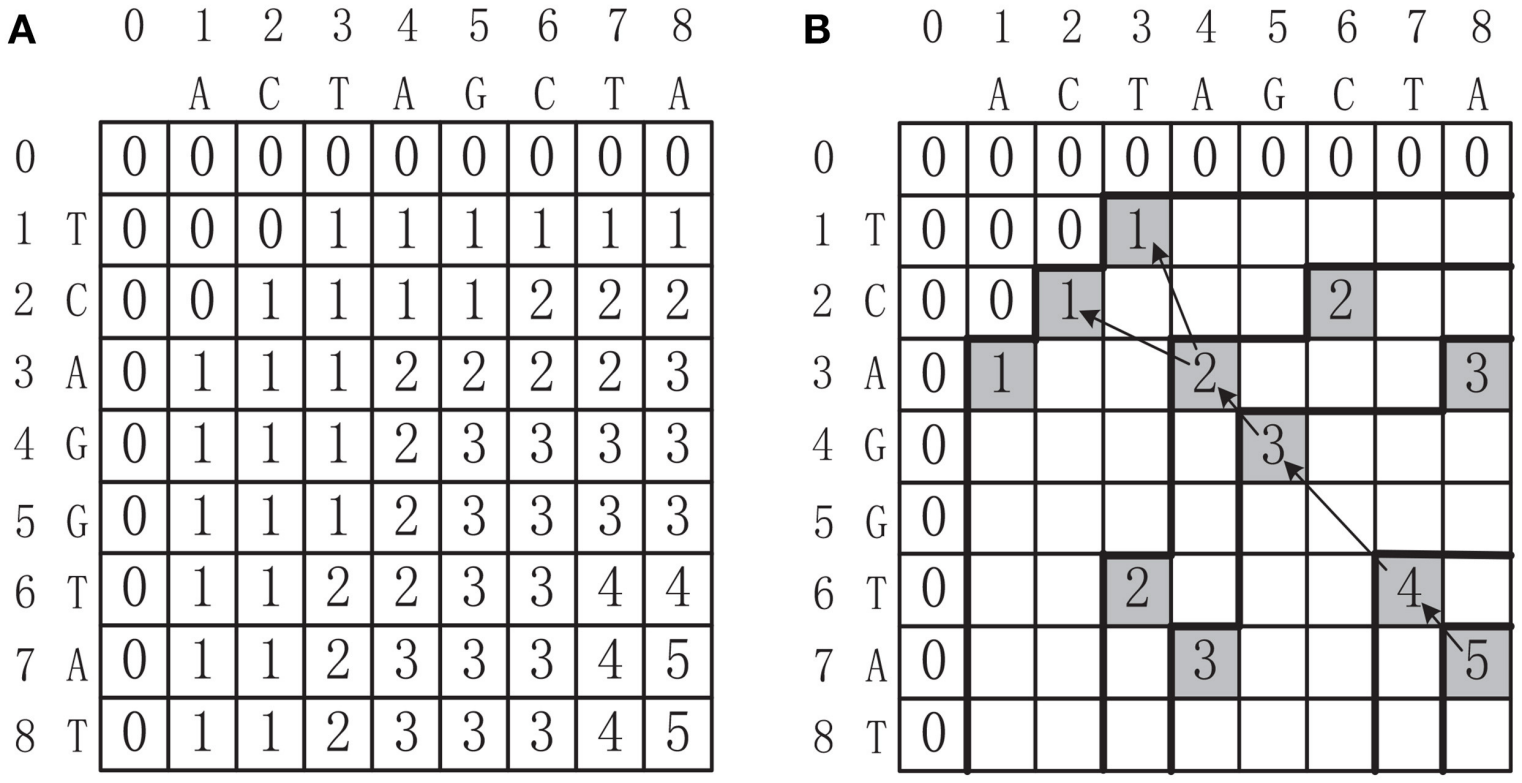
**(A)** The score table of two DNA sequences ACTAGCTA and TCAGGTAT. **(B)** Constructing the MLCS from the score table, where the shaded cells conspond to dominant points.

The classical approaches for the MLCS problem are based on dynamic programming (Sankoff, 1972; Smith and Waterman, 1981). Given *d* sequences *s*_1_, *s*_2_, …, *s*_*d*_ of length *n*_1_, *n*_2_, …, *n*_*d*_, respectively, these approaches recursively construct a score table *T* having *n*_1_ × *n*_2_ × … × *n*_*d*_ cells, in which the cell *T*[*i*_1_, *i*_2_, …, *i*_*d*_] records the length of MLCS of the prefixes *s*_1_[1…*i*_1_], *s*_2_[1…*i*_2_], …, *s*_*d*_[1…*i*_*d*_]. Specifically, *T*[*i*_1_, *i*_2_, …, *i*_*d*_] can be computed recursively by the following formula:

(1) $$\begin{array}{l} T\left[i_{1},i_{2},\ldots ,i_{d}\right]\\ \;=\{\begin{array}{ll} 0 & \text{if }\exists j(1\leq j\leq d),i_{j}=0\\ T[i_{1}-1,\ldots ,i_{d}-1]+1 & \text{if }s_{1}[i_{1}]=s_{2}[i_{2}]=\ldots =s_{d}[i_{d}]\\ max(\bar{T}) & \text{otherwise} \end{array} \end{array}$$

where $\overset{-}{T}=\left\{T\left[i_{1}-1,i_{2},\ldots ,i_{d}\right],T\left[i_{1},i_{2}-1,\ldots ,i_{d}\right],\ldots ,T\left[i_{1},i_{2},\ldots ,i_{d}-1\right]\right\}$. Once the score table *T* is constructed, the MLCS can be collected by tracing back from the last cell *T*[*n*_1_, *n*_2_, …, *n*_*d*_] to the first cell *T*[0, 0, …, 0]. Figure 1A shows the score table *T* of two sequences *s*_1_ = *ACTAGCTA* and *s*_2_ = *TCAGGTAT*. The MLCS of these two sequences, which are *TAGTA* and *CAGTA*, can be found by tracing back from *T*[8, 8] to *T*[0, 0], as shown in Figure 1B.

Obviously, both time and space complexity of dynamic programming based approaches for a MLCS problem with *d* sequences of length *n* are *O*(*n*^*d*^) (Hsu and Du, 1984). Many methods have been proposed to improve the efficiency, Hirschberg (1977), Apostolico et al. (1992), Masek and Paterson (1980), and Rick et al. (1994). However, with the increase of *d* and *n*, all these approaches are still inefficient from practical use.

#### 2.1.2. Dominant point based approaches

In order to reduce the time and space complexity of the dynamic programming based approaches, many other methods have been proposed, among which the dominant point based approaches are the most efficient ones until now. Before discussing the dominant point based approaches, some related definitions are introduced first:

**Definition 3**. Given *d* sequences *s*_1_, *s*_2_, …, *s*_*d*_ on Σ, a vector *p* = (*p*_1_, *p*_2_, …, *p*_*d*_) is called a *match point* of the *d* sequences, if *s*_1_[*p*_1_] = *s*_2_[*p*_2_] = … = *s*_*d*_[*p*_*d*_] = δ, i.e., δ is a common symbol appearing at the position *p*_*i*_ of sequence *s*_*i*_ for *i* = 1, 2, ⋯ , *d*. The corresponding symbol δ of match point *p* is denoted by *C*(*p*).

**Definition 4**. Given two match points *p* = (*p*_1_, *p*_2_, …, *p*_*d*_) and *q* = (*q*_1_, *q*_2_, …, *q*_*d*_) of *d* sequences, we call: (1) *p* = *q* if and only if *p*_*i*_ = *q*_*i*_ (1 ≤ *i* ≤ *d*). (2) *p dominates q* (or *q* is *dominated* by *p*), which is denoted by *p* ≼ *q*, if *p*_*i*_ ≤ *q*_*i*_ for each *i* (1 ≤ *i* ≤ *d*), and *p*_*j*_ < *q*_*j*_ for some *j* (1 ≤ *j* ≤ *d*). (3) *p strongly dominates q* (or *q* is *strongly dominated* by *p*), which is denoted by *p* ≺ *q*, if *p*_*i*_ < *q*_*i*_ for each *i* (1 ≤ *i* ≤ *d*). (4) *q* is a *successor* of *p* (or *p* is a *precursor* of *q*), if *p* ≺ *q* and there is no match point *r* such that *p* ≺ *r* ≺ *q* and *C*(*q*) = *C*(*r*).

Not that, one match point can have at most |Σ| successors with each successor corresponding to one symbol in Σ.

**Definition 5**. The *level* of a match point *p* = (*p*_1_, *p*_2_, …, *p*_*d*_) is defined to be *L*(*p*) = *T*[*p*_1_, *p*_2_, …, *p*_*d*_], where *T* is the score table computed by Formula (1). A match point *p* is called a *k-dominant point* (*k-dominant* for short) if and only if: (1) *L*(*p*) = *k*. (2) There is no other match point *q* such that: *L*(*q*) = *k* and *q* ≼ *p*. All the *k*-dominants form a set *D*^*k*^.

The motivation of the dominant point based approaches is to reduce the time and space complexity of the basic dynamic programming based methods. The key idea is based on the observation that only the dominant points can contribute to the construction of the MLCS (as shown in Figure 1B, the shaded cells correspond to the dominant points). Since the number of dominant points can be much smaller than the number of all cells in the score table *T*, a dominant point approach that only identifies the dominant points, without filling the whole score table, can greatly reduce the time and space complexity.

The search space of the dominant point based approaches can be organized to a directed acyclic graph (DAG): a node in DAG represents a match point, while an edge 〈*p, q*〉 in DAG represents that *q* is a successor of *p*, i.e., *p* ≺ *q* and *L*(*q*) = *L*(*p*) + 1. Initially, the DAG contains only a *source node* (0, 0, …, 0) with no incoming edges as well as an *end node* (∞, ∞, …, ∞) with no outgoing edges. Next, the DAG is constructed level by level as follows: at first, let the level *k* = 0, and *D*^0^ = {(0, 0, …, 0)}, and then, with a forward iteration procedure, the (*k* + 1)-dominants *D*^*k* + 1^ are computed based on the *k*-dominants *D*^*k*^, and this procedure is denoted by *D*^*k*^ → *D*^*k* + 1^. Specifically, each node in *D*^*k*^ is expanded by generating all its |Σ| successors, then a pruning operation called *Minima* is performed to identify those successors who dominant others, and only those dominants are reserved to *D*^*k* + 1^. Once all the nodes in the graph have been expanded, the whole DAG is constructed, in which a longest path from the source node to the end node corresponds to a LCS, thus, the MLCS problem becomes finding all the longest paths from the source node to the end node. In the following, we will use a simple example to illustrate the above procedure.

**Figure 2:**
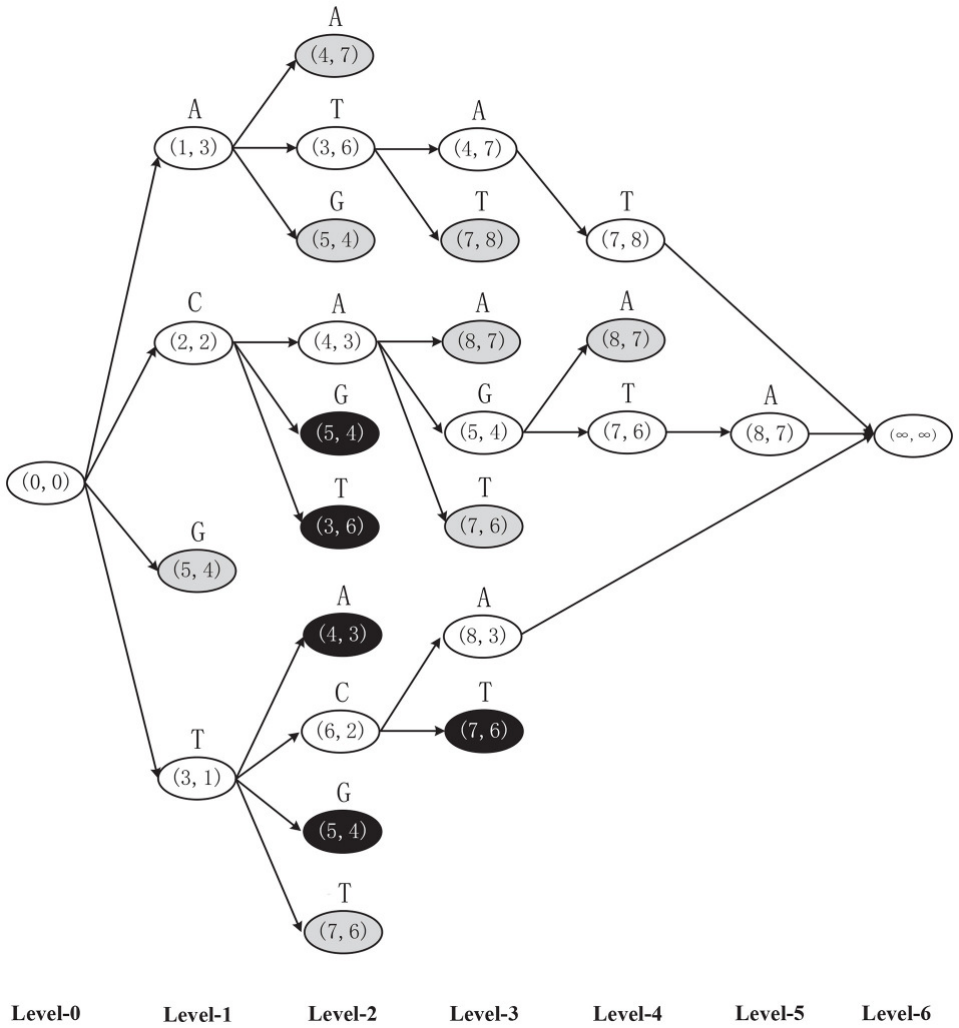
The DAG of two sequences *ACTAGCTA* and *TCAGGTAT* constructed by the general dominant point based algorithms, in which the black and gray nodes will be eliminated by the *Minima* operation.

**Example 1**. Finding the MLCS of sequences *ACTAGCTA* and *TCAGGTAT* based on the dominant point based approaches, as shown in Figure 2.

- **Step 0**. Set source node (0, 0) and end node (∞, ∞).
- **Step 1**. Construct nodes in level 1. For symbol *A*, the components of match point (1, 3) are the first positions of *A* in the two input sequences from beginning. Thus, node *A*(1, 3) is a successor of the source node corresponding to symbol *A* in level 1. Similarly, node *C*(2, 2), *G*(5, 4), and *T*(3, 1) are also the successors of the source node corresponding to symbol *C*, *G* and *T*, respectively. Among these four nodes in level 1, find and delete dominated node *G*(5, 4) (using the *Minima* operation), as shown in gray in Figure 2. The left three dominant nodes form *D*^1^ = {*A*(1, 3), *C*(2, 2), *T*(3, 1)}.
- **Step 2**. Construct nodes in level 2. For each node in *D*^1^, e.g., for *A*(1, 3)∈*D*^1^, symbol *A* with match point (4, 7) is the first common symbol *A* in the two sequences after symbol *A* with match point (1, 3) (i.e., after node *A*(1, 3)∈*D*^1^). Thus, node *A*(4, 7) is a successor of *A*(1, 3) corresponding to symbol *A* in level 2. Similarly, nodes *T*(3, 6) and *G*(5, 4) are also successors of *A*(1, 3) corresponding to symbol *T* and *G*, respectively, in level 2. In the same way, node *C*(2, 2) in level 1 can generate three successors *A*(4, 3), *G*(5, 3) and *T*(3, 6) in level 2, and node *T*(3, 1) in level 1 can generate four successors *A*(4, 3), *C*(6, 2), *G*(5, 4), and *T*(7, 6) in level 2. Note that, some nodes appear more than one times. Among these ten nodes in level 2, find and delete the duplicated nodes (using *Minima*) *A*(4, 3), *G*(5, 4) (delete two times) and *T*(3, 6) as shown in black in level 2. Also, find and delete all dominated nodes (using *Minima*) (4, 7), (5, 4), and (7, 6) as shown in gray in level 2. The left dominant points form the set *D*^2^ = {*T*(3, 6), *A*(4, 3), *C*(6, 2)}, which forms the final level 2 of the graph. Note that, if a node has no successors, let the end node to be its only successor.
- **Step 3**. Repeat the above construction process level by level until the whole DAG is constructed.

It can be seen from the above example that the dominant point based approaches have the following main drawbacks:

1. There are a huge number of duplicated nodes and dominated nodes in each level, which consumes a lot of memory.
2. All these duplicated nodes and dominated nodes in each level should be deleted, and finding all these nodes in each level needs a lot of pairwise comparisons of two *d* dimensional vectors (while each pairwise comparison of two *d* dimensional vectors needs *d* pairwise comparisons of two integers). Thus, the deletions of duplicated nodes and dominated nodes in all levels will be very time consuming.

Hunt and Szymanski (1977) proposed the first dominant point based algorithm for two sequences with time complexity *O*((*r* + *n*)*logn*), where *r* is the number of nodes in DAG and *n* is the length of the two sequences. Afterwards, to further improve the efficiency, a variety of dominant point based LCS/MLCS algorithms have been presented. Korkin (2001) proposed the first parallel MLCS algorithm with time complexity *O*(|Σ||*D*|), where |*D*| is the number of dominants in the graph. Chen et al. (2006) presented an efficient MLCS algorithm—FAST-LCS for DNA sequences, it introduced a novel data structure called successor table to obtain the successors of nodes in constant time and used a pruning operation to eliminate the non-dominant nodes in each level. Wang et al. (2011) proposed an algorithm Quick-DPAR to improve the FAST-MLCS algorithm, it uses a divide-and-conquer strategy to eliminate the non-dominant nodes, which is very suitable for parallelization, it is indicated that the parallelized algorithm Quick-DPPAR had gained a near-linear speedup compared to its serial version. Li et al. (2012) and Yang et al. (2010) made efforts to develop efficient parallel algorithms on GPUs for the LCS problem and on cloud platform for the MLCS problem, respectively. Unfortunately, Yang et al. (2010) is not suitable for the MLCS problem with many sequences due to the large synchronous costs. Recently, Li et al. (2016b,a) proposed two algorithms: PTop-MLCS and RLP-MLCS based on dominant points, these algorithms used a novel graph model called Non-redundant Common Subsequence Graph (NCSG) which can greatly reduce the redundant nodes during processing, and adopted a two-passes topological sorting procedure to find the MLCS. The authors claimed that the time and space complexity of their algorithms is linear to the number of nodes in NCSG.

In practice, for MLCS problems with large number of sequences, the traditional algorithms usually need a long time and large space to find the optimal solution (the complete MLCS), to address this issue, approximate algorithms have been investigated to quickly produce a suboptimal solution (partial MLCS) and gradually improve it when given more time, until an optimal one is found. Yang et al. (2013) proposed an approximate algorithm Pro-MLCS as well as its efficient parallelization based on the dominant point model. Pro-MLCS can find an approximate solution quickly, which only takes around 3% of the entire running time, and then progressively generates better solutions until obtaining the optimal one. Recently, Yang et al. (2014) proposed another two approximate algorithms SA-MLCS and SLA-MLCS. SA-MLCS used an iterative beam widening search strategy to reduce space usage during the iterative process of finding better solutions. Based on SA-MLCS, SLA-MLCS, a space-bounded algorithm, is developed to avoid space usage from exceeding the available memory.

### 2.2. A new graph model: Leveled-DAG and its construction algorithm

In this section, we introduce the Leveled-DAG model as well as its construction algorithm. Before describing the details, we first introduce some key data structures used.

#### 2.2.1. Key data structures

I. Successor table

**Figure 3:**
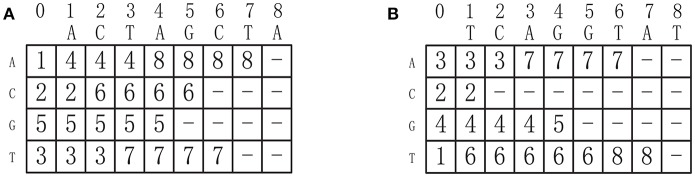
**(A)** The successor table of sequence ACTAGCTA. **(B)** The successor table of sequence TCAGGTAT.

Efficiently generating the successors of a node is a key operation in constructing Leveled-DAG. For this purpose, we need to construct a *successor table* (Chen et al., 2006) for each input sequence. Through the successor tables, we can generate the successors of each node in constant time. Specifically, given a sequence *s* = *c*_1_*c*_2_…*c*_*n*_, the corresponding successor table (denoted by *ST*) of *s* is a two-dimensional array with |Σ| × (*n* + 1) elements, in which the element of the *i*-th row and *j*-th column of *ST* (denoted by *ST*[*i, j*]) is defined as follows:

(2) $$\begin{array}{l} ST\left[i,j\right]=min\left\{m|c_{m}=\sigma_{i},m>j,1\leq i\leq |\Sigma |,0\leq j\leq n\right\} \end{array}$$

where σ_*i*_ is the *i*-th symbol in Σ. Indeed, *ST*[*i, j*] specifies the position of the first occurrence of symbol σ_*i*_ in *s*, starting from the (*j* + 1)-sort position. For instance, the successor tables of two DNA sequences *ACTAGCTA* and *TCAGGTAT* are shown in Figures 3A,B, respectively. Given *d* sequences of length *n*, we can construct all the corresponding successor tables in *O*(*d*|Σ|*n*) time.

By referring to the successor tables, given *d* sequences, all the successors of one node can be generated in *O*(*d*|Σ|) time. For example, the successors of the node C(2, 2) in Figure 2 can be obtained by referring the successor tables shown in Figure 3: (*ST*_1_[1, 2], *ST*_2_[1, 2]) = (4, 3), (*ST*_1_[2, 2], *ST*_2_[2, 2]) = (6, −), (*ST*_1_[3, 2], *ST*_2_[3, 2]) = (5, 4) and (*ST*_1_[4, 2], *ST*_2_[4, 2]) = (3, 6), corresponding to *A*, *C*, *G*, and *T*, respectively, where (6, −) means (2, 2) has no successor corresponding to *C*. In fact, a node can have no successors at all.

II. The structure of the node in Leveled-DAG

For each node, say *t*, in Leveled-DAG, it mainly contains the following information:

- The match point of *t*, which is regarded as the unique identifier of *t*.
- *Suc*(*t*): the set of all *successors* of *t*.
- *P*_*LCS*(*t*): the set of all partial LCSs corresponding to the longest paths from the source node to *t*.

The match point of a node *t* is used to identify whether *t* has existed in the graph. As will be seen later, whenever a node is deleted, its partial LCSs will be inherited and extended by its successors. Once the construction of Leveled-DAG is completed, only the end node is left in the graph, and the *P_LCS* of the end node contains the wanted MLCS of the input sequences.

III. Global data structures

- *L_DAG* : the data structure to maintain the nodes in the graph.
- *Cur*_*Level* : a queue to store the current level of nodes in the graph.
- *Next*_*Level* : a queue to store the (newly generated) next level of nodes in the graph.

The *L*_*DAG* is a mapping table to hold the generated nodes. A node can be found in *L*_*DAG* by its key (i.e., the mach point), and whenever a new node is created, we search its match point in *L*_*DAG* to check whether the node is already existed, if not, we insert that node into *L*_*DAG*. The queue *Cur*_*Level* is used to store the nodes to be expanded, and the queue *Next*_*Level* is used to store the newly created successors.

#### 2.2.2. The new graph model: Leveled-DAG

The key feature of the Leveled-DAG model is that it adopts a strategy called *generation and deletion* to control the scale of the graph. Specifically, once a new level of nodes are created, all the nodes in the graph with no incoming edges are *outdated* and will be deleted, because they can no longer be successors of any subsequent node and their partial LCSs will not be changed any more. Therefore, they will not affect the construction of MLCS in the following procedure when they are deleted. Thus, timely deleting these *outdated* nodes will greatly reduce the scale of graph and save a lot of memory. By using this strategy, the Leveled-DAG is constructed level by level from the source node, and at any moment, only the nodes in current level and the nodes with incoming edge in the previous level are kept in memory. Moreover, once the construction is finished, only the end node is left in the graph, and the wanted MLCS of the input sequences are saved in the end node, thus, no additional operations for searching the MLCS are needed, which can save a lot of time.Next, we will give an example to illustrate the Leveled-DAG model.

**Figure 4:**
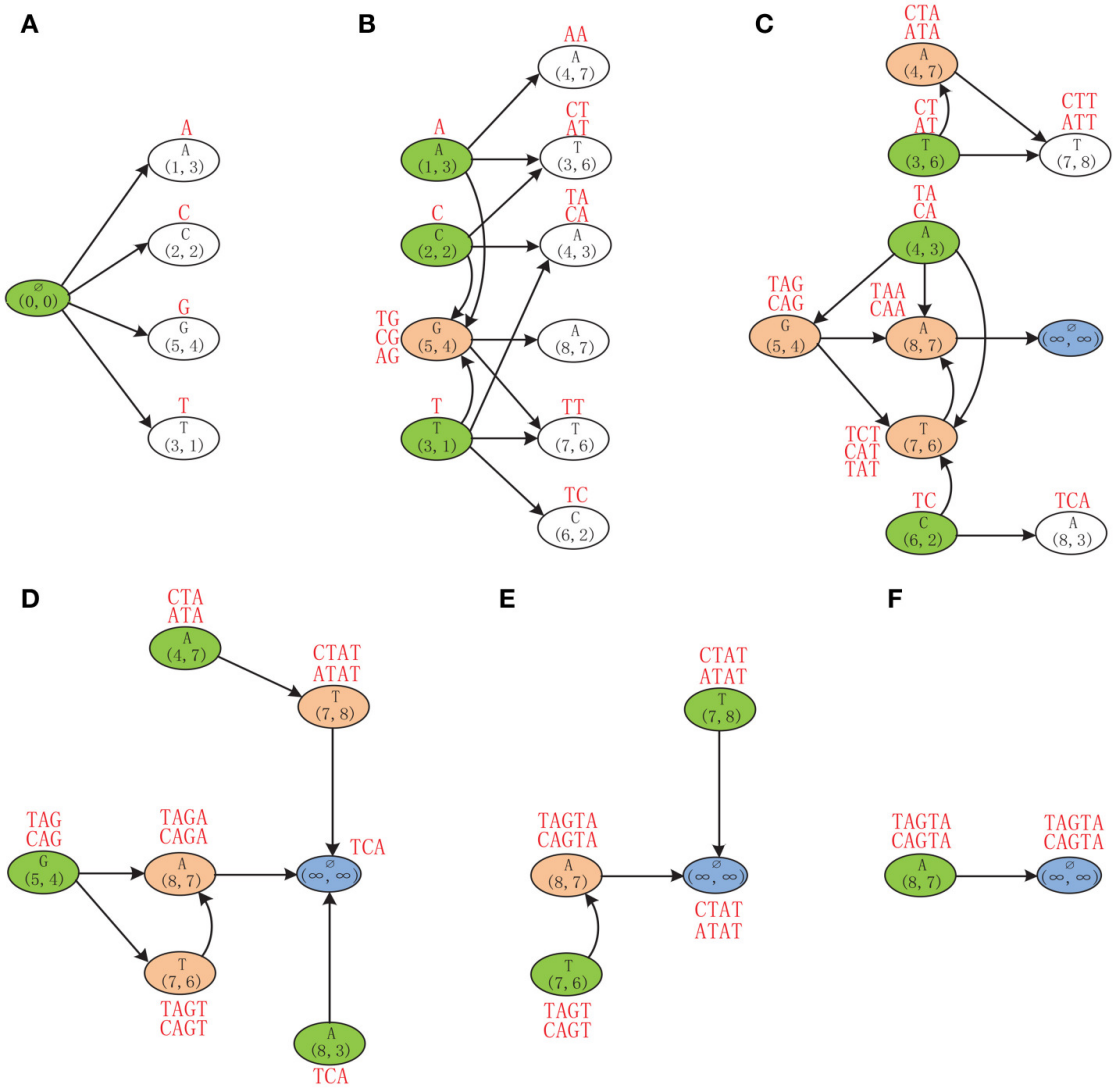
The Leveled-DAG constructed for sequences ACTAGCTA and TCAGGTAT. The mach point and the corresponding symbol are shown in each node. The partial LCSs are shown by red strings near the nodes. The white nodes are newly created and will be expanded later. The green ones are outdated and will be removed right away. The red ones with incoming edges are left from the previous levels and cannot be removed at present. **(A)** Generate the first level of nodes. **(B)** Generate the second level of nodes. **(C)** Generate the third level of nodes. **(D)** No new node is created any more. **(E)** Delete the remaining outdated nodes. **(F)** Only the end node is left.

**Example 2**. Finding the MLCS of sequences *ACTAGCTA* and *TCAGGTAT* based on the Leveled-DAG model. Initially, the Leveled-DAG contains only the source node (0, 0) in level 0. Then the successors of the source node, which are *A*(1, 3), *C*(2, 2), *G*(5, 4), and *T*(3, 1) corresponding to the four symbols, respectively, are generated by referring to the successor tables (Figure 3). Because the four successors are in level 1, their partial LCSs are single characters *A*, *C*, *G*, and *T*, respectively, as shown by the red symbols above the corresponding nodes in Figure 4A. After that, since the source node has no incoming edges, it is outdated and can be safely deleted from the graph.

Then, generate the successors of nodes in the first level as the second level of nodes, which are *A*(4, 7), *T*(3, 6), *A*(4, 3), *A*(8, 7), *T*(7, 6), and *C*(6, 2), as shown in Figure 4B. Note that, if a successor has already existed in Leveled-DAG (such as *G*(5, 4)), it needs not to be repeatedly created. After the second level of nodes have been created, since *A*(1, 3), *C*(2, 2), and *T*(3, 1) have no incoming edges, they are outdated and should be deleted, also, their partial LCSs should be inherited and extended by their successors. For instance, the outdated node *A*(1, 3) has three successors (it has no successor corresponding to *C*): *A*(4, 7), *T*(3, 6), and *G*(5, 4). Each of these successors needs to append its own corresponding symbol to the partial LCS of *A*(1, 3) (which is *A*), and then saves that appended partial LCS as its own partial LCS. Specifically, the successor *A*(4, 7) appends *A* to *A*, and saves *AA* as its partial LCS. The successor *T*(3, 6) appends *T* to *A*, and saves *AT* as its partial LCS. Similarly, the successor *G*(5, 4) appends *G* to *A*, and saves *AG* as its partial LCS. Note that, since *G*(5, 4) is the successor of all three outdated nodes, it gets three partial LCSs, which are *TG*, *CG*, and *AG*, by inheriting and extending the partial LCSs of its three outdated precursors. After removing the outdated nodes, only 7 nodes are left in the graph.

Next, as shown in Figure 4C, the nodes in the second level are expanded by generating their successors, and the newly created successor nodes *T*(7, 8) and *A*(8, 3) form the third level of the graph. Note that, since *A*(8, 7) has no successors, the end node (∞, ∞) is defined to be its only successor. After constructing the third level, the nodes *T*(3, 6), *A*(4, 3), and *C*(6, 2) become outdated. Before removing them, their partial LCSs will be inherited and extended by their successors first. As a special case, since *A*(4, 7) is a successor of the outdated node *T*(3, 6), the partial LCSs of *T*(3, 6), which are *CT* and *AT*, will be inherited by *A*(4, 7) and appended by *A*. Because the appended partial LCSs *CTA* and *ATA* are longer than the original partial LCS of *A*(4, 7) (which is *AA*), the partial LCSs of *A*(4, 7) will be updated to *CTA* and *ATA* accordingly. Similarly, the partial LCSs of *G*(5, 4) and *T*(7, 6) are also updated by inheriting and extending the partial LCSs of their outdated precursors.

As shown in Figure 4D, the newly generated nodes *T*(7, 8) and *A*(8, 3) in the third level of the graph are expanded by generating their successors. Since neither of them has successors, the end node is defined to be the successor of both nodes. Because no new node is created, no node needs to be expanded any more. Therefore, from this moment on, the algorithm only needs to repeatedly remove the outdated nodes and update the partial LCSs of their successors. At present, *A*(4, 7), *G*(5, 4), and *A*(8, 3) will be removed. By inheriting the partial LCS of *A*(8, 3), the end node gets *TCA* as its partial LCS (since the end node corresponds to no symbol, nothing is appended to *TCA*). Next, as shown in Figure 4E, *T*(7, 6) and *T*(7, 8) will be removed, and the partial LCSs of the end node are updated to *CTAT* and *ATAT*. Finally, as shown in Figure 4F, after removing the last outdated node *A*(8, 7), the partial LCSs of end node are updated to *TAGTA* and *CAGTA*, which are the wanted MLCS of the two input DNA sequences.

From the example we can see that, at the beginning, only the source node resides in Leveled-DAG, and then the number of nodes grows gradually, once there is no new node created, the number of nodes begin to decline until only one node is left. During the procedure, only the new created nodes and the nodes with incoming edges are saved in Leveled-DAG, which greatly reduce the memory consumption. Once the construction is finished, the MLCS can be obtained immediately.

#### 2.2.3. Construction algorithm for the Leveled-DAG model

In this section, we will give a formal description of the construction algorithm for the Leveled-DAG model.

**Algorithm 1. Construction of Leveled-DAG**

- **Step 0**. Preprocessing. For each input sequence, construct its successor table.
- **Step 1**. Construct the first level of Leveled-DAG. Generate all successors of the source node as the first level by referring to the successor tables. Let the corresponding symbol of each new successor to be its single char partial LCS. Delete the source node.
- **Step 2**. Construct the next level of Leveled-DAG, and delete the outdated nodes (*generate and delete*). If there exists nodes in Leveled-DAG that have not been expanded, repeat the following two sub-steps:
  – **Step 2.1**. For each node *t* that is not expanded, generate all successors of *t* (if a certain successor has already existed in the graph, it does not need to be generated repeatedly and just needs to make a pointer to it), and if *t* has no successors, let the end node be its only successor.
  – **Step 2.2**. Let |*p*| denote the length of the partial LCSs of node *p*. For each node *p* that has no incoming edges, and for each successor *s* of *p*:
    * If |*p*| ≥ |*s*|, delete the (old) partial LCSs of *s*. Append the corresponding symbol of *s* to each partial LCS of *p*, and then save all the appended partial LCSs as the new partial LCSs of *s*.
    * Otherwise, if |*p*| = |*s*|−1, append the corresponding symbol of *s* to each partial LCS of *p*, and add all the appended partial LCSs to the existing partial LCSs of *s*.
  Delete node *p* (as well as its partial LCSs) from the graph.
- **Step 3**. Repeat Step 2.2, until only the end node is left in the graph.
- **Step 4**. Output the partial LCSs saved in the end node, which are the real MLCS of the input sequences.

**Algorithm 1:** Pseudocode

**Input:**
The successor tables of the input sequences.
**Output:**
The MLCS of the input sequences.

*Suc*(*source*) ← ∅, *P*_*LCS*(*source*) ← ∅
*Suc*(*end*) ← ∅, *P*_*LCS*(*end*) ← ∅
*L*_*DAG* ← {*source, end*}
*Cur*_*Level* ← {*source*}

**while** *Cur*_*Level* ≠ ∅ **do**
**for** each node *t* ∈ *Cur*_*Level* **do**
**for** each successor *s* of *t* **do**
*Suc*(*t*) ← *Suc*(*t*)∪{*s*}
**if** *s* ∉ *L*_*DAG* **then**
*L*_*DAG* ← *L*_*DAG*∪{*s*}
*Next*_*Level* ← *Next*_*Level*∪{*s*}
**end if**
**end for**
**if** *t* has no successor **then**
*Suc*(*t*) ← {*end*}
**end if**
**end for**
*Remove*_*Outdated*(*L*_*DAG*)
*Cur*_*Level* ← *Next*_*Level*
**end while**

**while** ∃*t* ∈ *L*_*DAG* and *t* ≠ *end* **do**
*Remove*_*Outdated*(*L*_*DAG*)
**end while**

**Output**: *P*_*LCS*(*end*)

*Remove*_*Outdated*(*L*_*DAG*):
**for** each node *p* ∈ *L*_*DAG* that has no incoming edges **do**
**for** each successor *s* of *p* **do**
|*p*| ← the length of partial LCSs of *p*
|*s*| ← the length of partial LCSs of *s*
δ ← the corresponding symbol of *s*
**if** |*p*| ≥ |*s*| **then**
**for** each *plcs* ∈ *P*_*LCS*(*p*) **do**
Append δ to *plcs*
**end for**
*P*_*LCS*(*s*) ← {all the appended partial LCSs}
**else if** |*p*| + 1 = |*s*| **then**
**for** each *plcs* ∈ *P*_*LCS*(*p*) **do**
Append δ to *plcs*
**end for**
*P*_*LCS*(*s*) ← *P*_*LCS*(*s*) ∪ {all the appended partial LCSs}
**end if**
Delete *p* from *L*_*DAG*
**end for**
**end for**

As shown in **Algorithm 1**, after preprocessing (Step 0), the Leveled-DAG contains only the source node at the beginning (Step 1), and then it adopts a *generate and delete* strategy to generate the next level of Leveled-DAG (Step 2.1) and delete the outdated nodes (Step 2.2), once all the nodes in Leveled-DAG have been expanded, i.e., there is no new node created, the algorithm begins to repeatedly delete the outdated nodes (Step 3), until only the end node is left. At last, output the partial LCSs of the end node, which are the MLCS of the input sequences (Step 4).

The pseudocode of **Algorithm 1** is shown above, where the data structures used are introduced in Section 2.2.1. The preprocessing procedure of building the successor tables is omitted in the pseudocode, and the successor tables are used directly as input data. The Step 1 and Step 2 in **Algorithm 1** are combined into a big *while* loop of line 7 ~ 22, and Step 3 corresponds to a *while* loop of line 24 ~ 26. The operations to remove the outdated nodes in Leveled-DAG are wrapped into a single procedure called *Remove*_*Outdated* corresponding to line 30 ~ 49.

#### 2.2.4. Analysis of time and space complexities

Next, we will give a rough estimate of the time and space complexities of the Leveled-DAG approach.

As mentioned above, the procedure of Leveled-DAG approach consists of two stages: 1. Build the successor table for each input sequence; 2. Construct the Leveled-DAG graph based on the successor tables. For the first stage, as shown in Section 2.2.1, building the successor table for a given sequence of length *n* takes *O*(|Σ|*n*) time, thus, the time complexity of building all the successor tables for *d* input sequences of length *n* is *O*(*d*|Σ|*n*). For the second stage, from the overall point of view, construction of Leveled-DAG is just generating all the nodes of the graph and then deleting them all. Although the generating and deleting procedures are mixed together during running, one node is generated and deleted only once, and there are no recursive procedures at all (as shown in the pseudocode code of **Algorithm 1**). Therefore, the time complexity of constructing the Leveled-DAG is *O*(2|*Nodes*|), where |*Nodes*| is the number of all nodes created, and each of the generating and deleting procedure takes *O*(|*Nodes*|) time. Combine the two stages, the time complexity of the Leveled-DAG approach is *O*(*d*|Σ|*n* + 2|*Nodes*|). Since there is always *O*(*d*|Σ|*n*) ≪ *O*(2|*Nodes*|) (this is indicated by experiments), we have *O*(*d*|Σ|*n* + 2|*Nodes*|) ≈ *O*(2|*Nodes*|) ≈ *O*(|*Nodes*|), which means the time complexity of the Leveled-DAG approach is linear to the number of nodes in the graph.

For the space consumption, on the one side, our approach needs to store all the successor tables which takes *O*(*d*|Σ|(*n* + 1)) space. On the other side, as stated before, the Leveled-DAG graph (mainly) needs to store the latest level of nodes, and the number of nodes in the latest level will increase first and then decline, thus the memory consumption of the Leveled-DAG graph will grow to a peak and then decrease. Therefore, the (peak) space consumption of the Leveled-DAG graph will be *O*(|*Max*_*Level*|), where |*Max*_*Level*| is the number of nodes in the max level of the graph. Combine the two sides, the space complexity of Leveled-DAG approach is *O*(*d*|Σ|(*n* + 1) + |*Max*_*Level*|), and since there is always *O*(*d*|Σ|(*n* + 1)) ≪ *O*(|*Max*_*Level*|), we have *O*(*d*|Σ|(*n* + 1) + |*Max*_*Level*|) ≈ *O*(|*Max*_*Level*|), which means the space complexity of the Leveled-DAG approach depends on the max level of the graph.

## 3. Results

In this section, we compare the time and space efficiency of the Leveled-DAG approach with three other state-of-the-art algorithms: *Top_MLCS* by Li et al. (2016b), *Quick-DP* by Wang et al. (2011) and *Fast_LCS* by Chen et al. (2006) on real biological sequences.

### 3.1. Experimental setups

We choose two types of biological sequences: DNA sequences with |Σ| = 4 and protein sequences with |Σ| = 20 as the input data. We conduct two kinds of experiments:

1. Evaluation under various numbers of sequences: for each type of the sequences, the number of sequences used increases from 3 to 700 and the length of all used sequences is fixed to 100.
2. Evaluation under various lengths of sequences: for each type of the sequences, the number of sequences is fixed to 5, but the length of the sequences increases from 50 to 5,000.

For each test, according to the specified number and length, the input sequences are generated by randomly extracting and truncating a large raw sequence set. In the experiments, all the test algorithms are run on a server with Intel Xeon E7-8880 2.2 GHz CPU and 700 GB RAM (since the server is shared by many users, the available memory for each process is at most 300 GB). The operating system is GNU/Linux (amd64), and all the algorithms are implemented with C/C++ and compiled by *gcc* with option “−O2.”

### 3.2. Evaluation under various numbers of sequences

In the first kind of experiment, each test is conducted on a specific number of sequences from 3 to 700 with the length fixed to 100, for both DNA and protein sequences. All the algorithms are independently run 5 times with 32 threads, and their average running times (as well as the standard deviation of the running times) and memory consumption are measured and shown in Tables 1, 2, respectively. It is worth pointing out that, the running time and the memory consumption of the algorithms are highly depended on the contents of test sequences. In particular, for the sequence sets with the same size but containing different sequences, the time and memory consumption of the test algorithms may vary significantly.

**Table 1:** The average running times (in seconds) of the test algorithms on different numbers of DNA and protein sequences with the length of sequences fixed to 100. (Using 32 threads).

**Number**	**DNA (|Σ| = 4)**				**Protein (|Σ| = 20)**			
	**FAST_LCS**	**Quick-DP**	**Top_MLCS**	**Leveled-DAG**	**FAST_LCS**	**Quick-DP**	**Top_MLCS**	**Leveled-DAG**
3	0.052 (0.003)	0.041 (0.001)	0.031 (0.002)	0.018 (0.001)	0.021 (0.001)	0.034 (0.002)	0.027 (0.001)	0.016 (0.001)
4	0.255 (0.01)	0.203 (0.02)	0.071 (0.004)	0.053 (0.003)	0.183 (0.02)	0.152 (0.03)	0.051 (0.003)	0.037 (0.002)
5	2.9 (0.1)	1.5 (0.09)	0.12 (0.008)	0.082 (0.004)	2.1 (0.1)	1.0 (0.08)	0.098 (0.008)	0.077 (0.006)
6	26.5 (1.7)	10.3 (0.8)	1.3 (0.09)	1.1 (0.1)	20.8 (1.2)	6.9 (0.6)	0.94 (0.03)	0.75 (0.02)
7	151.8 (10.0)	32.8 (1.9)	3.6 (0.1)	2.7 (0.2)	116.5 (10.3)	21.8 (1.3)	2.8 (0.2)	1.9 (0.1)
8	834.9 (43.8)	147.6 (8.8)	8.5 (0.6)	6.9 (0.4)	746.5 (58.8)	107.0 (8.7)	6.3 (0.3)	4.7 (0.2)
9	4,174.6 (408.7)	738.5 (51.5)	16.4 (0.9)	13.7 (0.6)	3,059.2 (301.4)	585.6 (40.2)	12.5 (0.8)	9.9 (0.4)
10	25,671.4 (2,433.8)	3,385.4 (326.4)	30.0 (2.3)	25.3 (0.9)	22,751.9 (1,596.7)	2,645.3 (215.7)	24.7 (1.5)	20.6 (1.1)
20	–	–	64.8 (4.6)	51.2 (2.1)	–	–	57.7 (3.3)	45.3 (2.3)
30	–	–	136.7 (6.7)	96.7 (3.7)	–	–	124.3 (8.9)	89.2 (4.7)
40	–	–	250.3 (9.4)	191.4 (7.5)	–	–	223.4 (14.7)	180.8 (10.5)
50	–	–	463.2 (17.5)	380.1 (12.3)	–	–	432.7 (22.4)	366.4 (16.0)
60	–	–	665.4 (42.2)	530.3 (27.4)	–	–	590.6 (31.2)	509.5 (20.4)
70	–	–	1,088.1 (76.7)	875.5 (39.4)	–	–	967.8 (76.4)	848.6 (39.6)
80	–	–	1,684.6 (127.5)	1,233.2 (62.3)	–	–	1,432.6 (105.2)	1,167.0 (66.8)
90	–	–	2,217.9 (188.3)	1,764.6 (85.5)	–	–	2,053.5 (127.1)	1,715.1 (84.1)
100	–	–	3,041.5 (220.9)	2,417.8 (174.9)	–	–	2,320.2 (144.8)	2,056.2 (101.5)
200	–	–	3,398.3 (241.4)	2,778.2 (190.2)	–	–	2,492.6 (152.4)	2,118.5 (114.9)
300	–	–	3,665.0 (263.8)	2,962.5 (206.7)	–	–	2,614.2 (165.7)	2,214.3 (138.8)
400	–	–	3,981.6 (285.0)	3,191.2 (218.0)	–	–	2,745.3 (172.8)	2,375.4 (152.9)
500	–	–	4,237.2 (310.3)	3,384.0 (231.4)	–	–	2,862.4 (181.1)	2,435.1 (164.3)
600	–	–	4,555.9 (336.9)	3,547.2 (243.7)	–	–	2,947.9 (193.4)	2,479.2 (170.7)
700	–	–	4,880.3 (362.7)	3,854.7 (266.2)	–	–	3,174.8 (204.5)	2,511.9 (183.2)

*The standard deviations of the running times are shown in the parentheses*.

**Table 2:** The memory consumption (in MB) of the test algorithms on different numbers of DNA and protein sequences with the length of sequences fixed to 100.

**Number**	**DNA (|Σ| = 4)**				**Protein (|Σ| = 20)**			
	**FAST_LCS**	**Quick-DP**	**Top_MLCS**	**Leveled-DAG**	**FAST_LCS**	**Quick-DP**	**Top_MLCS**	**Leveled-DAG**
3	28	31	8	5	25	28	7	4
4	373	447	23	17	330	403	19	14
5	1,358	1,485	93	85	1,167	1,304	77	62
6	3,315	3,490	297	223	2,718	2,960	203	190
7	5,190	5,862	534	489	4,152	47,06	469	418
8	11,057	12,051	1,211	1,124	8,513	9,871	1,017	943
9	20,634	21,183	3,058	2,765	15,138	16,062	2,538	2,238
10	35,769	36,934	5,813	5,232	25,637	26,048	4,766	4,251
20	–	–	32,329	28,126	–	–	24,246	18,045
30	–	–	48,765	39,291	–	–	36,824	26,713
40	–	–	67,813	52,607	–	–	49,503	35,182
50	–	–	91,128	68,103	–	–	64,292	46,137
60	–	–	121,268	87,359	–	–	81,379	58,174
70	–	–	156,470	118,600	–	–	98,541	61,036
80	–	–	197,387	141,859	–	–	117,390	76,283
90	–	–	209,145	146,402	–	–	120,833	81,429
100	–	–	229,372	151,386	–	–	124,124	84,069
200	–	–	252,247	163,948	–	–	131,920	88,132
300	–	–	261,963	167,085	–	–	138,255	92,025
400	–	–	268,993	170,811	–	–	144,213	96,044
500	–	–	276,103	173,945	–	–	151,318	101,250
600	–	–	290,398	177,140	–	–	157,986	104,986
700	–	–	299,498	179,846	–	–	162,298	108,107

The experimental results show that, the *FAST_LCS* and *Quick-DP* algorithms cannot process datasets containing 20 (or more) sequences, due to out of memory. As shown in Table 2, the memory consumption of these two algorithms are quite close (since they use the same framework and the only difference is how to delete the dominated points) and both grow exponentially as the number of sequences increases, because both algorithms need to generate a huge number of redundant nodes and save them all in memory. As shown in Table 1, their running times also grow rapidly as the number of sequences increases, and this is mainly because that, as the number of sequences increases, the dimension of the match point in each node increases accordingly. Therefore, to eliminate the dominated points, the *Minima* operation used by both algorithms needs to compare each pair of the match points dimension by dimension at every level. This needs a lot of comparisons and is extremely time-consuming. On the other hand, *Quick-DP* is much more efficient than *FAST_LCS* since it adopts a *divide and conquer* strategy to eliminate the dominated points, which is very suitable for parallelization. Unfortunately, the *Quick-DP* algorithm is still not efficient enough in either time or space for sets with many sequences.

By contrast, it can be seen from the results that the *Top_MLCS* algorithm and the proposed *Leveled-DAG* algorithm are able to process up to 700 sequences. As shown in Table 2, the memory consumptions of these two algorithms are much less than that of the previous ones, this is because both algorithms adopt new graph models to reduce the scale of the dominant point graph: the *ICSG* model adopted by *Top_MLCS* will not generate the redundant nodes, while the *Leveled-DAG* model only keeps one level (and some more) nodes of the graph in memory. Compared with *Top_MLCS*, the *Leveled-DAG* algorithm can save about 35% ~ 40% / 33% ~ 35% of the space for DNA/protein sequence sets with more than 100 sequences, this is due to that for large sequence sets the nodes saved by *Leveled-DAG* (which includes the max level of nodes as well as the nodes retained from the previous levels) account for only about 40% of the total nodes. Also note that, at the beginning, the memory consumptions of both algorithms grow rapidly as the number of sequences increases, but after the sequences increasing to a certain volume (about 80 ~ 90 in this experiment) the memory growth rate begins to decline, and finally the growth of memory trends to be roughly a constant. We find that this is because the increase of nodes begins to slow down once the volume of sequences exceeds a certain threshold (the threshold is hard to determine since it depends on many factors such as the alphabet of sequences, the length of sequences and the contents of sequences), and finally the number of nodes remains roughly the same for further increase of sequences, and at this time the memory growth mainly comes from the increase of the dimension of match point in each node.

Moreover, both algorithms are significantly (about one to two orders of magnitude) faster than *FAST_LCS* and *Quick-DP*. This mainly because they do not need a similar operation such as the *Minima* operation used by *FAST_LCS* and *Quick-DP* to make comparisons on each pair of match point. Compared with *Top_MLCS*, our *Leveled-DAG* is about 10% faster for smaller datasets (containing less than 10 sequences), and 10% ~ 20% faster for larger datasets. This is because the *Top_MLCS* algorithm needs two topological sorting operations (forward and backward topological sort) to search for the MLCS after the graph is built, while the *Leveled-DAG* does not need any searching operation after *Leveled-DAG* is constructed. In fact, the wanted MLCS are saved in the only end node left in the graph, which can be gotten immediately. In summary, the *Leveled-DAG* approach is more suitable than the compared algorithms for large-scale sequences sets. Note that, the growth in running time of both algorithms also begins to slow down as the number of sequences grows to a threshold.

From Tables 1, 2, we can also see that both time and space efficiency of all the test algorithms for protein sequences are superior to that for DNA sequences, this is because the scale of DAG for large alphabet sequences (such as protein) is smaller than that for small alphabet sequences (such as DNA). We leave the detailed discussion of the effect of alphabet size in the later section.

### 3.3. Evaluation under various lengths of sequences

In the second kind of experiment, each test is conducted on 5 DNA/protein sequences with length increasing from 50 to 5,000. As before, all the algorithms are independently run 5 times with 32 threads, and their average running times (as well as the standard deviation of the running times) and memory consumption are measured and shown in Tables 3, 4, respectively.

**Table 3:** The average running times of the test algorithms under different lengths of DNA and protein sequences with the number of sequences fixed to 5. (Using 32 threads).

**Length**	**DNA (|Σ| = 4)**				**Protein (|Σ| = 20)**			
	**FAST_LCS**	**Quick-DP**	**Top_MLCS**	**Leveled-DAG**	**FAST_LCS**	**Quick-DP**	**Top_MLCS**	**Leveled-DAG**
50	0.57 (0.03)	0.13 (0.01)	0.038 (0.002)	0.026 (0.001)	0.06 (0.001)	0.018 (0.001)	0.004 (0)	0.001 (0)
100	2.7 (0.2)	1.4 (0.08)	0.23 (0.03)	0.96 (0.04)	0.3 (0.02)	0.16 (0.01)	0.077 (0.003)	0.058 (0.006)
200	244.1 (10.4)	10.6 (0.2)	8.5 (0.3)	6.8 (0.2)	28.5 (1.5)	1.2 (0.1)	0.96 (0.102)	0.77 (0.05)
300	4,064.8 (312.6)	95.3 (4.7)	38.7 (2.2)	32.6 (2.7)	467.1 (14.4)	11.4 (1.1)	4.4 (0.2)	3.1 (0.2)
400	–	312.4 (11.5)	77.8 (4.9)	59.5 (3.8)	3,659.2 (363.8)	36.7 (1.8)	8.9 (0.8)	7.2 (0.6)
500	–	1,566.9 (128.9)	132.6 (7.8)	112.2 (5.6)	–	180 (6.2)	15.2 (1.1)	12.7 (0.9)
600	–	4,384.1 (297.4)	201.1 (11.7)	165.3 (8.3)	–	533.8 (19.4)	23.1 (2.0)	18.9 (1.0)
700	–	10,347.5 (913.2)	287.3 (12.1)	223.4 (10.1)	–	1,075.5 (32.7)	32.6 (2.5)	24.6 (1.03)
800	–	27,489.2 (2,351.3)	373.2 (14.5)	313.8 (13.3)	–	2,958.1 (61.5)	43.9 (3.8)	35.3 (1.1)
900	–	–	487.3 (21.6)	399.1 (15.5)	–	6,709.0 (221.2)	54.1 (4.2)	44.8 (1.2)
1,000	–	–	644.7 (29.3)	513.5 (19.8)	–	11,508.6 (1,258.9)	70.8 (5.7)	57.0 (1.8)
2,000	–	–	4,240.5 (251.2)	3,017.6 (87.2)	–	–	469.3 (13.7)	355.4 (10.2)
3,000	–	–	9,915.1 (673.8)	7,922.0 (297.3)	–	–	1,168.5 (30.5)	873.1 (22.4)
4,000	–	–	16,963.4 (1,553.3)	13,762.3 (1,433.4)	–	–	1,843.0 (41.3)	1,532.5 (32.4)
5,000	–	–	24,672.9 (2,104.3)	19,074.7 (1,658.1)	–	–	2,788.3 (55.6)	2,065.2 (46.3)

*The standard deviations of the running times are shown in the parentheses*.

**Table 4:** The memory consumption (in MB) of the test algorithms under different lengths of DNA and protein sequences with the number of sequences fixed to 5.

**Length**	**DNA (|Σ| = 4)**				**Protein (|Σ| = 20)**			
	**FAST_LCS**	**Quick-DP**	**Top_MLCS**	**Leveled-DAG**	**FAST_LCS**	**Quick-DP**	**Top_MLCS**	**Leveled-DAG**
50	47	56	21	17	40	42	18	11
100	1,352	1,481	99	82	1,163	1,296	81	56
200	8,331	8,652	2,353	1,469	6,249	7,963	1,894	988
300	16,874	16,993	4,050	3,051	11,047	12,735	3,251	1,864
400	–	27,355	5,866	4,787	113,665	20,586	4,819	3,012
500	–	41,257	8,297	6,654	–	32,771	6,770	4,351
600	–	60,912	12,063	8,598	–	46,009	9,023	5,806
700	–	85,733	18,550	10,163	–	65,574	11,652	7,513
800	–	126,483	26,070	14,250	–	86,684	14,725	9,426
900	–	–	36,341	20,539	–	111,748	18,380	11,573
1,000	–	–	49,442	27,985	–	140,457	22,507	13,690
2,000	–	–	95,784	55,549	–	–	45,633	27,811
3,000	–	–	152,178	86,732	–	–	71,058	42,669
4,000	–	–	224,135	125,454	–	–	99,564	58,937
5,000	–	–	301,375	165,756	–	–	134,568	77,502

It can be seen from the experimental results that, the *FAST_LCS* cannot process DNA sequences longer than 400 or protein sequences longer than 500 due to the extremely long running time, while the *Quick-DP* algorithm cannot process DNA sequences longer than 800 or protein sequences longer than 1,000 due to out of memory. (Again, the performance of the algorithms for protein sequences is better than that for DNA sequences). Since as the length of the sequences increases, the number of levels in the graph will grow accordingly and the nodes in each level will grow exponentially, which makes the graph take up too much memory. Also, the running times of *FAST_LCS* and *Quick-DP* grow rapidly as the length of sequences increases, the main reason is that as the nodes increase exponentially in each level, the *Minima* operation for the levels is very time-consuming, moreover searching the longest paths in a graph with many levels is also time-consuming. Consequently, neither of the two algorithms are suitable for finding MLCS of long sequences.

On the other hand, as shown in Table 4, both *Top_MLCS* and *Leveled-DAG* can handle DNA/ protein sequences with length up to 5,000. Compared with *Top_MLCS*, the *Leveled-DAG* approach can save about 43% ~ 46% / 41% ~ 45% of memory for DNA/protein sequences longer than 1,000, since the growth in memory requirement of *Leveled-DAG* mainly depends on the max level of nodes, whose proportion in the total nodes will decrease as the sequence length increases. Further, as shown in Table 3, the running times of both algorithms grow much more slowly than that of *FAST_LCS* and *Quick-DP*. Even for long sequences (*length* ≥ 1, 000), both *Top_MLCS* and *Leveled-DAG* can still find their MLCS. Particularly, note that in all cases, the proposed algorithm *Leveled-DAG* is the fastest algorithm: it is at least two orders of magnitude faster than *FAST_LCS* and *Quick-DP*, and faster than *Top_MLCS* about 20% on datasets with longer sequences (*length* ≥ 20, 00). The reason for the proposed algorithm *Leveled-DAG* being faster than *Top_MLCS* on longer sequences is that with the increase of the length of sequences, the first topological sorting scheme used in *Top_MLCS* will take much more time, while the performance of *Leveled-DAG* will not be affected much by the sequence length. Thus *Leveled-DAG* algorithm is more suitable for finding MLCS of long sequences.

In general, due to the much smaller scale of the *Leveled-DAG* graph and the efficiency of the technique to gradually construct the MLCS, the *Leveled-DAG* approach has better performance than the compared algorithms on all the testing datasets, especially on datasets with long sequences and many sequences.

## 4. Discussion

Next, we will discuss some factors that can affect the performance of the MLCS algorithms.The *length* of the sequences is the key factor that affects the performance of the algorithms: for the same type of sequences, as the length of sequences increases, the number of levels in the corresponding DAG will increase accordingly. Since the number of nodes in the levels will grow (nearly) exponentially as the level increases, the total number of nodes in DAG will explode as the sequence length increases, therefore, the scale of DAG for long sequences is larger than that for short sequences. Consequently, both time and space overhead for finding MLCS of long sequences are higher than that for finding MLCS of short sequences. On the other hand, the *number* of sequences can also have an impact on the algorithms' performance: as the number of sequences increases, the dimension of match point in each node will grow accordingly, and therefore each single node in DAG will take up more space, moreover, comparing two match point will require more time. In addition, the number and length of the sequences can also affect the result MLCS: obviously, the longer the sequences are, the longer the result MLCS are; conversely, the more the sequences there are, the shorter the result MLCS are.

From the experimental results, we can also find that, the alphabet size can have a significant impact on the performance of the algorithms. For sequences with large alphabet (such as protein sequences), the performance of the algorithms is much better than that for sequences with small alphabet (such as DNA sequences). This is because that, for a fixed-length sequence, the larger the alphabet size is, the less each symbol appears in that sequence, which means for large alphabet sequences, the “distance” between one node and its successors is large, therefore the DAG for large alphabet sequences has less levels than that for small alphabet sequences. For instance, with the sequence length fixed to 100, the DAG for DNA sequences has about 30 levels, while the DAG for protein sequences has only about 10 levels. Although a certain level in DAG for protein sequences has more nodes than the same level in DAG for DNA sequences (since one node in DAG for protein sequences can have up to 20 successors, however, for DNA sequences one node has at most 4 successors), the total number of graph nodes for protein sequences is smaller than that for DNA sequences. Therefore, the performance of algorithms for large alphabet sequences is much better than that for small alphabet sequences.

By profiling the program, we find that the most time-consuming part of the Leveled-DAG approach is the procedure of deleting the outdated nodes (i.e., the *Remove*_*Outdated*(*L*_*DAG*) routine in the pseudocode of **Algorithm 1**, which takes about 50% ~ 60% of the total running time), particularly, the procedure of passing the partial LCSs of one node to its successors. This procedure needs a lot of memory allocation, resize, and free operations, which is not appropriate for parallelization and therefore very time-consuming. To reduce the time consumption, we are focusing on more efficient passing strategy and more carefully implementation. We are also searching for more efficient memory management routines to replace the ones we used (which are provided by the standard library).

## 5. Conclusion

In this paper, we propose a novel graph model called Leveled-DAG which is much smaller than the existing DAG model. Based on this model, the corresponding construction algorithm is also proposed. Once the Leveled-DAG is constructed, there is only one node left with all MLCS being immediately obtained without any further operation for searching the MLCS. The experimental results also show the proposed approach is both time and space efficient on all testing cases, especially for long and large-scale sequences.

## Author contributions

ZP designs and implements the proposed algorithm. ZP writes the initial manuscript, and YW revises the manuscript.

### Conflict of interest statement

The authors declare that the research was conducted in the absence of any commercial or financial relationships that could be construed as a potential conflict of interest.

## References

[B1] Aravanis A. M. Lee M. Klausner R. D. (2017). Next-generation sequencing of circulating tumor dna for early cancer detection. Cell 168, 571–574. 10.1016/j.cell.2017.01.030 28187279

[B2] Apostolico A. Browne S. Guerra C. (1992). Fast linear-space computations of longest common subsequences. Theor. Comput. Sci. 92, 3–17. 10.1016/0304-3975(92)90132-Y

[B3] Chen Y. Wan A. Liu W. (2006). A fast parallel algorithm for finding the longest common sequence of multiple biosequences. BMC Bioinformatics 7(Suppl. 4):S4. 10.1186/1471-2105-7-S4-S4 17217522 PMC1780122

[B4] Hirschberg D. S. (1977). Algorithms for the longest common subsequence problem. J. ACM 24, 664–675. 10.1145/322033.322044

[B5] Hsu W. J. Du M. W. (1984). Computing a longest common subsequence for a set of strings. BIT Numer. Math. 24, 45–59. 10.1007/BF01934514

[B6] Hunt J. W. Szymanski T. G. (1977). A fast algorithm for computing longest common subsequences. Commun. ACM 20, 350–353. 10.1145/359581.359603

[B7] Korkin D. (2001). A New Dominant Point-based Parallel Algorithm for Multiple Longest Common Subsequence Problem. Technical report, Technical report, Department of Computer Science, University of New Brunswick.

[B8] Li Y. Li H. Duan T. Wang S. Wang Z. Cheng Y. (2016a). A real linear and parallel multiple longest common subsequences (mlcs) algorithm, in Proceedings of the 22Nd ACM SIGKDD International Conference on Knowledge Discovery and Data Mining, KDD '16 (New York, NY: ACM), 1725–1734. 10.1145/2939672.2939842

[B9] Li Y. Wang Y. Bao L. (2012). FACC: a novel finite automaton based on cloud computing for the multiple longest common subsequences search. Math. Prob. Eng. 2012:310328 10.1155/2012/310328

[B10] Li Y. Wang Y. Zhang Z. Wang Y. Ma D. Huang J. (2016b). A novel fast and memory efficient parallel MLCS algorithm for long and large-scale sequences alignments, in 2016 32nd IEEE International Conference on Data Engineering (ICDE) (Helsinki: IEEE), 1170–1181. 10.1109/ICDE.2016.7498322

[B11] Maier D. (1978). The complexity of some problems on subsequences and supersequences. J. ACM 25, 322–336. 10.1145/322063.322075

[B12] Masek W. J. Paterson M. S. (1980). A faster algorithm computing string edit distances. J. Comput. Syst. Sci. 20, 18–31. 10.1016/0022-0000(80)90002-1

[B13] Rick C. Rick C. Rick C. (1994). New Algorithms for the Longest Common Subsequence Problem. Technical report.

[B14] Sankoff D. (1972). Matching sequences under deletion/insertion constraints. Proc. Natl. Acad. Sci. U.S.A. 69, 4–6. 10.1073/pnas.69.1.4 4500555 PMC427531

[B15] Smith T. Waterman M. (1981). Identification of common molecular subsequences. J. Mol. Biol. 147, 195–197. 10.1016/0022-2836(81)90087-5 7265238

[B16] Wang Q. Korkin D. Shang Y. (2011). A fast multiple longest common subsequence (MLCS) algorithm. IEEE Trans. Knowl. Data Eng. 23, 321–334. 10.1109/TKDE.2010.123 PMC4231498 25400485

[B17] Yang J. Xu Y. Shang Y. (2010). An efficient parallel algorithm for longest common subsequence problem on gpus, in Proceedings of the world congress on engineering, WCE, vol. 1 (London, UK).

[B18] Yang J. Xu Y. Shang Y. Chen G. (2014). A space-bounded anytime algorithm for the multiple longest common subsequence problem. IEEE Trans. Knowl. Data Eng. 26, 2599–2609. 10.1109/TKDE.2014.2304464 25400485 PMC4231498

[B19] Yang J. Xu Y. Sun G. Shang Y. (2013). A new progressive algorithm for a multiple longest common subsequences problem and its efficient parallelization. IEEE Trans. Parall. Distribut. Syst. 24, 862–870. 10.1109/TPDS.2012.202

[B20] Zvelebil M. Baum J. (2007). Understanding Bioinformatics. Garland Science—Taylor & Francis Group.

